# A Local-Exchange Model of Folding Chain Surface of Polymer Crystal Based on Worm-Like Chain Model within Single-Chain in Mean-Field Theory

**DOI:** 10.3390/polym12112555

**Published:** 2020-10-30

**Authors:** Hongyi Xiao, Xinghua Zhang, Dadong Yan

**Affiliations:** 1 Department of Physics, Beijing Normal University, Beijing 100875, China; 16121620@bjtu.edu.cn; 2 School of Science, Beijing Jiaotong University, Beijing 100044, China

**Keywords:** worm-like chain, loop brush, folding surface of polymer crystal

## Abstract

The structure of amorphous layer of folding surface controls the properties of the polymer lamellar crystal, which consists of chains with a loop conformation. The surface tension depends on the length and the distance between two injection points of the loop which involving the reptation motion and lateral exchange motion of the stems. In the present work, a local-exchange motion model based on the worm-like chain model is developed to investigate the effects of lateral motion of stems on the release the surface tension. The optimal distance between two injection points is determined by the balance of chain bending energy and conformational entropy. The numerical results provide evidences to the adjacent re-entry model for various loop lengths. A possible explanation involving density of injection points is proposed to interpret the mechanism.

## 1. Introduction

Polymer crystallization is one of the most important issues in polymer science. Two-thirds of common polymer materials can be crystallized. Controlling the degree of crystallization is the major way to tune the performances of polymer materials, including their mechanical properties, photoelectric properties, thermal conductivity, etc. The mechanism of polymer crystallization are extensively studied. The chain-connectivity makes the problem complex. Although the experimental phenomena have been reported, and the comprehensive theoretical description about polymer crystallization is scarce [1]. In ideal crystal formed by small molecules, molecules are arranged on an infinite periodic lattice. The polymer chains in their perfect crystals, should be straightened fully, and packed parallelly. This kind of polymer crystal is called as infinite extended chain crystal. Nevertheless, it is impossible for actual polymer chains to organize their configurations in this way to form the perfect crystal in a finite period. With this constrains, the polymer forms folded chain crystal (also called the lamellar crystal) generally [2,3,4]. The stems whose lengths are much shorter than the total contour lengths of polymer chains consist the lamellar crystals. The folded chain segments form the amorphous layers.

In the 1960s, Hoffman and Lauritzen proposed their theory of polymer crystallization, which afterwards was called classical theory [5,6,7,8,9]. This theory treats the crystallization behavior as a successive process that the stems attach one by one onto the growth surface. They derived the spontaneous selection of lamellar thickness and the growth rate, and agree with the experimental results in their age [10]. The prerequisite of this theory is the existing primary nucleus (or a lamellar crystal already exists). In this theory the lamellar has two surface: one is the lateral surface on which the stem deposition happens, and another is the chain-folding surface. In fact, the growth of lamellar crystals both the adhesion of stem on the lateral surface and the increase lamellar thickness by the regulating the conformation of folding chains in amorphous layers. The Lauritzen–Hoffman (L-H) theory pays attention to the successive quasi-equilibrium processes on the lateral surface. Its structure and roles on crystallization have not well addressed.

The folding chain surfaces consist of loops, and a small amount of bridges and tails [11]. Around this theme there are two contrary mainstream models, adjacent re-entry model proposed by Keller [2,12] and revised by Fischer [3], and switchboard model suggested by Flory [13,14]. The former supposes the segments in non-growth surface layer re-enter into the lamellar crystal at the sites which are nearby the location where they project out from the crystal region, while the later supposes the re-entries happen at the sites with arbitrary distances with the projecting-out points. The adjacent re-entry model also divides into two typical schematism. The first one is Keller’s original model [2] which assumes that the loops re-enter the crystal region after experiencing a short length in interface layer, to form a tight loop. The second one is Fischer’s revised model [3], which describes the loops with a remarkable longer length, i.e., loose loops. Some other variants or revisions of the models about non-growth surfaces are developed successively, while their key ideas are contained in the above-mentioned models.

Muthukumar used Langevin dynamics and Monte Carlo method to simulate the polymer crystallization to study the primary nucleation, the spontaneous selection of lamellar thickness, and the growth kinetics [15,16]. The loop-like conformation formed by chain-folding plays an important role in these aspects. First, the folding surface tension is mainly contributed by the entropy penalty due to formation of the loop conformation. An optimal fraction of chain segments in the crystal can be determined from the balance between free energy gain due to forming the crystal and the entropy penalty due to forming the loop conformation. The entropy penalty can be estimated analytically when two injection points of the loops are assumed to be fixed. This theory had agreed with several experiments. In this theory the distance between two injection points of a loop is assumed to be a constant. This parameter is a length scale that should be optimized. How the injection points are arranged on the surface, and how the loops are packed in the amorphous left are still unclear.

Muthukumar’s theory with an explicit chain model for the amorphous layers, was extended from single-chain system to the multi-chain system by Sommer [17]. The results indicate that the lamellar thickness increases with the number of chains in the crystal, and extended chain crystals are formed if the number of chains in the crystal is large enough (more than the square of the degree of polymerization of the chains in thermodynamic equilibrium states). Sommer’s multi-chain crystal model relies on two main approximations, i.e., the Gaussian chain statistics and the flat, non-fluctuating folding chain surface. He investigated the loops with finite bending rigidity and the surface with a tilt angle, respectively [18]. Moreover, it has been pointed that the free energy per loop increases substantially as the end-to-end distance of loops become larger, and reveals the preference of tight loops and close re-entries qualitatively under experimental condition below equilibrium melting temperature. A monotonically increasing relation between free energy per loop and end-to-end distance of loops is obtained by this theory. This is because the bending energy of the chain was ignored. Besides, the quantitative optimal value and the distribution of end-to-end distance are still undetermined.

Both Muthukumar’s theory and Sommer’s model indicated that an optimal distance between two injection points exists, which allows the rearrangement of stems and loops after the polymer segments are crystallized. Because two ends of a loop are linked to two stems in lamellar crystal, loops can change their end-to-end distance by the lateral motion of stems, and then minimize the surface tension.

According to the simulation of growth of the lamellar, there are two motion modes: the lateral motion and the parallel motion. The lateral motion (which will be called local-exchange in this paper) is the stem moving in the lateral direction in its entirety. The parallel motion is the reptation motion of the stem, which involves the monomers moving in and out the crystal region. Both motion modes change the loop conformations. The lateral motion changes the end-to-end distance of the loop and the parallel motion change the length of the loops. The simulation work indicated that the lateral motion continues throughout the crystallization process and is even more important than parallel motion during the early stage [19,20]. This is just what the L-H theory does not answer. L-H theory is a secondary nucleation theory, in which primary nucleation is already finished [10].

The folding structure of polymer chains in the amorphous layers was observed by Savage et al. recently [21] using torsional trapping atom force microscopy (AFM). Both many first-neighbor folds and a smaller number of second-neighbor folds are caught. The simulations of interface region can be traced back to Balijepalli’s work in the 1990s [22,23,24,25]. He modelled the interface layer consisting of loops, tails and bridges by freely rotating chain [22,24], and tilt of surface is considered [25]. Nilsson et al. added two kinds of conformations, free chains and entangled loops, based on Balijepalli’s model, and the hindered rotation model was applied [26]. The SCFT is introduced to investigate the interface structure by Milner [27] and Shah [28]. Because the density of ends is rather low in long polymer systems, Milner modelled the interface by a homogeneous loop-brush in half-space without tail and bridge [27]. Shah et al considered the crystallization of multi-block copolymers constituted by components with various rigidity. Lempesis et al. simulated the interface between the crystalline and amorphous domains of poly(tetramethylene oxide) by molecular dynamics [11]. In general, these theoretical models including loops with variable length correspond to parallel motion model. On the other hand, a theoretical model including loops with variable end-to-end distance corresponds to the lateral motion model. Balijepalli gave the density profile of re-entry sites [22], and Sommer derived the qualitative preference of small end-to-end distance under the equilibrium conditions [17].

The polymeric loop brush is a helpful theoretical model to characterize the conformation of polymers in the amorphous layer [27]. The lamellar is considered as the substrate of the polymer brush. The injection points of a loop is modeled as the grafting points of the loop brush. The adjacent re-entry model and the switchboard model of crystallization can be modeled by continuously changing the distance between two injection points.

In the amorphous layer the length of loops are much shorter than the chain length and comparable with that of the Kuhn length. Therefore, its conformation deviates Gaussian statistic behavior and will be dominated by the semiflexible behavior. It has been reported that the end-to-end distance of the semiflexible chain depends on the chain end orientation and the chain length [29]. Therefore, the loop in the amorphous layer must has an optimal end-to-end distance. The surface tension of the amorphous will depend on the end-to-end distance.

In present work, the lateral motion model of amorphous layer based on the worm-like chain model has been put forward aiming to study the structure of the folding surface. In this model, the two injection points of the loop are allowed to move on the folding surface to model the local-exchange of the stems, which is different from the immobile loop brush [30], while the influence of the mobility of injection points is neglected. The single-chain in mean-field theory (SCMFT) is used to solve this model, in which the path integral of chain statistics in the auxiliary field is computed by the Monte Carlo simulation [31,32,33]. The conformations of the folded chain in the amorphous region can be well demonstrated by a Wang–Landau algorithm.

This paper is organized as follows: In Section 2 the model system and the numerical methods are provided which incorporate the Monte Carlo simulation, Wang—Landau algorithm and SCMFT; In Section 3 the main results of crystal-amorphous interfacial structure have been obtained; In Section 4 the discussion.

## 2. Theory and Numerical Method

### 2.1. Modeling the Crystal-Amorphous Interface

The amorphous layer is considered to be a polymer loop brush with two injection points connecting to two stems in the crystal. The crystal is modeled as the substrate of the brush and the stems are considered implicitly [10]. Considering stems exchange lateral position in the crystal, the free energy of the crystal region will not be changed. However, the free energy of the amorphous region will be changed due to the variation of the distance between injection points of loops. The parameters of interfacial “pseudo-brush” include (1) the contour length of loop chains, and (2) the areal density of “injected” chains. The latter concerns the lattice constant and is equivalent to “grafting density” in the language of polymer brushes. In above conditions, the system will determines the optimal distance between two injection points via minimizing chain bending and maximizing conformation entropy.

A minimum model is considered here to reveal the lateral motion of the stem to optimize the surface tension of the amorphous layer which is similar to Ref. [27]. According to the atomistic simulation of poly(tetramethylene oxide), the percentage of loops in the noncrystalline domain is the largest, and much larger than the sum of the corresponding number of bridges and tails [11], thus, the contribution of tails and bridges can be ignored. To simplify the study, we assume that the direction of stems is normal to the surface of lamellae, although this is not in full accord with the real situations. For example, both experiments [21,34] and simulations [11] point out that there is an angle between the stem and the normal direction of lamellar. Besides, Savage et al. also observed that the lamellae edges have a roughness in the order of nanometers [21,24,25]. The point we focus on here is the minimization of interfacial tension via changing the distance between two injection points. Considering the complexity of this problem, we fixed the loop length, and simplify the system as a mono-disperse loop brush. This simplification ignores the chain-slipping behavior, which will be taken into consideration in our future work.

**Figure 1:**
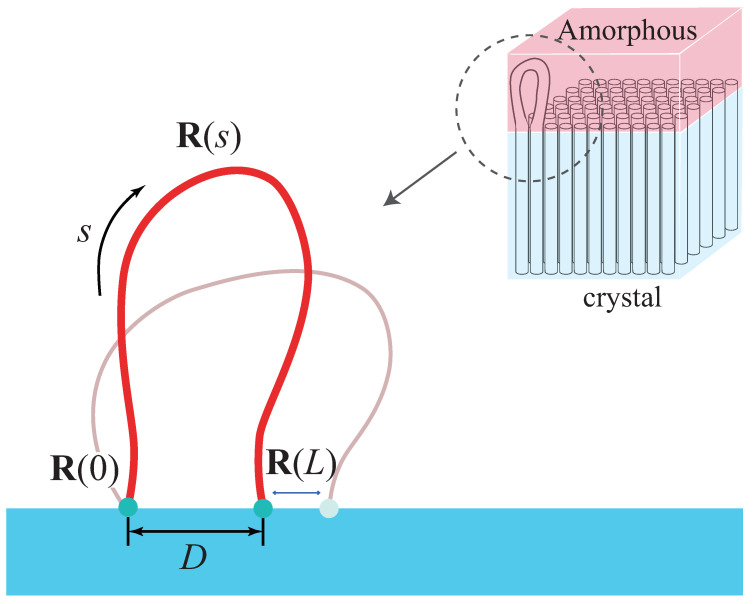
Schematic diagram of the local-exchange model for polymer crystal-amorphous interface.

We model the amorphous layer by *n* worm-like chains loops with their ends on the lamellar edge (the x−y plane at z=0) with area *A*, as shown in Figure 1. The lattice constant is *c*, and $c^{2}$ is inversely proportional to the density of injection points σ [17,18]. Each loop has a total contour length *L* with persistence length *a* and excluded diameter *d*. Henceforth, all the lengths are nondimensionalized by *a*. The configuration of loop is described by a spacial curve R(s), where s∈[0,L] is the contour variable. The distance between its two injection points is defined by D≡|R(L)−R(0)|. The interfacial free energy formula can be given by SCMFT (see Appendix A for details):

(1) $$\begin{array}{c} \beta F\left[\omega \right]\equiv -\frac{1}{2}\int drdudu^{\prime }\frac{\omega \left(r,u\right)\omega \left(r,u^{\prime }\right)}{2da^{2}\left|u\times u^{\prime }\right|}-n\ln Q\left[\omega \right], \end{array}$$

where u(s)=dR(s)/ds is the tangent vector of the chain. $\beta =1/k_{B}T$ with the Boltzmann factor $k_{B}$ and temperature *T*. ω is the auxiliary field. The Q[ω] term, which is the single chain partition function, can be written as function of *D* (see Appendix B for details):

(2) $$\begin{array}{c} -\ln Q\left(D\right)=-\ln\left\{g\left(D\right)\frac{\int_{0}^{E_{m}}dEg_{D,L}\left(E\right)\exp\left(-\beta E\right)}{\int_{0}^{E_{m}}dEg_{D,L}\left(E\right)}\right\}, \end{array}$$

where g(D), defined by Equation (A15), is the density of states for a given *D*; $g_{D,L}\left(E\right)$, defined by Equation (A21), is the density of energy states for a given *D* and *L*; $E_{m}$ is the ceiling energy. We design a modified Wang–Landau algorithm to accomplish the calculation of Q(D) in the ensemble [L,D,βE].

### 2.2. Modified Multi-Steps High-Precision Wang–Landau Algorithm

As two typical cases, L=1 and L=8 are chosen to represent the “tight fold” case and the “loose loop” case, respectively. Considering simple cubic polymer crystal, the density of injection points σ=n/A is inversely proportional to the square of lattice constant *c*, i.e., $\sigma =1/c^{2}$. As an example, in the following calculations, we take the persistence length a=2.5c at the crystallization temperature. With this parameter, σ=6.25 (i.e., $c=1/\sqrt{\sigma }\approx 0.4$). For a given auxiliary field ω, $5\times 10^{5}$ Monte Carlo steps are performed to make sure the chain equilibrates under the field and then $M∼10^{7}$ conformations are sampled to evaluate the ensemble average of density functional. Then we update the auxiliary field ω using the mean-field Equation (A8) with simple mixing algorithm [35] to reach the convergent result.

The ensemble average of any observable quantity 〈A〉 is computed by sampling the possible paths in the auxiliary field, ω, explicitly by Monte Carlo simulation, i.e.,

(3) $$\begin{array}{c} \langle A\rangle =\frac{1}{M}\sum_{j=1}^{M}A\left\{R_{j}\left(s\right)\right\}], \end{array}$$

where *M* is the total number of conformations sampled for the ensemble average and $R_{j}$ is the *j*-th conformation of a single chain in the auxiliary field sampled by the Metropolis algorithm.

The determination of g(D) written as Equation (A15) follows the algorithm proposed by Wang and Landau [36], except that the conventional reference parameter *E* is replaced by *D* [37]. The range of variation for D/L is within [0,1], where D/L=1 corresponds to the conformation which is fully stretched and clings to the two-dimensional lamellae edge. The D/L is evenly divided into m=100 equal bins, where the function g(D) for the *i*-th bin is represented by a variable $g_{i}\;\left(i=1,2,3...m\right)$. We take the densities of states $g_{i}$ equal to 1 and the accumulating histogram $H_{i}$ equal to 0 for all *i* in the beginning of procedure, together with a initial modification factor f=1.

The random walk in *D*-space is performed by to sample the conformation of inextend loop by Monte Carlo trial move without any consideration of energy. Two trial moves are used in present work: the lateral motion of the injection point and the crankshaft move. In the lateral motion, the segments between a randomly selected segment and a injection point are rotated by a random angle about the axis which is normal the substrate and goes through the selected segment. In the crankshaft move, the segments between randomly selected two segments are rotated by a random angle about the end-to-end vector of these segments. Both trial moves are subject to the hard-wall interaction of the substrate at z=0. The acceptance of the trial move from the conformation with $D_{1}$ to that with $D_{2}$ is $p\left(D_{1}\to D_{2}\right)=\min\left[g\left(D_{1}\right)/g\left(D_{2}\right),1\right]$.

After the Monte Carlo attempt, once the value of *D* visits the *i*-th bin, the density of state $\ln g_{i}$ is updated by the modification factor, i.e., $\ln g_{i}\leftarrow f+\ln g_{i}$, as well as the accumulating histogram updating by $H_{i}\leftarrow 1+H_{i}$. A simulation iteration terminates when the maximal difference $\left|H_{i}-\bar{H}\right|$, where $\bar{H}$ is the average of all $H_{i}$, is less than 1%. The modification factor, *f*, is then multiplied by 1/2, the histogram $H_{i}$ is reset to zero, and a new MC simulation iteration was conducted by using the new *f* until $f<10^{-9}$. An approximation for lng(D) within accuracy no more than $10^{-9}$ has been obtained.

The Wang–Landau algorithm was also used to estimate $g_{D,L}\left(E\right)$. In worm-like chain model, the upper bound of bending energy could be as high as $\beta E=10^{4}$ for L=1, corresponding to the zigzag conformation. However, the Boltzmann factor exp(−βE) would exceed the precision which the computer could achieve for βE>700. Considering the integrand is multiplied by $g_{D,L}\left(E\right)$ and exp(−βE), the precision of density of energy states near ground state is crucial because of the weighting of Boltzmann factor. The conventional Wang–Landau algorithm, could not meet the requirement of precision.

In the view of above-mentioned difficulty, we proposed a modified Wang–Landau scheme to determine the $g_{D,L}\left(E\right)$. It contains three steps:

(1) Giving a trial run of Wang–Landau algorithm in the whole energy space, aiming to search a configuration which can be treated as the ground state reasonably.

(2) The *E* space is divided into $N_{E}$ regions, and width of every region is η of that of the adjacent higher-energy region (in this paper, we take $N_{E}=12$ and η=0.6). Besides, each region has a small but enough part of overlap with its left and right neighbouring regions, respectively. Wang–Landau algorithm is independently performed in each energy region. A simulation iteration terminates when the maximal difference $\left|H_{i}-\bar{H}\right|$ is less than 1%, and the iterations of MC segments are considered convergent after *f* become smaller than $10^{-7}$.

(3) Up to present, the sectional $\ln g_{D,L}\left(E\right)$ in different energy regions had been obtained independently, but the difference between them is still unknown. To make all of them join together as a smooth curve, we“alining” the overlap of adjacent two regions, which means, shift $\ln g_{D,L}\left(E\right)$ of all regions till the overlap regions are coincide with their neighbors. This method manages visiting the energy states which are too low for a individual Wang–Landau run to explore.

## 3. Results and Discussion

### 3.1. Structure of Amorphous Layer

**Figure 2:**
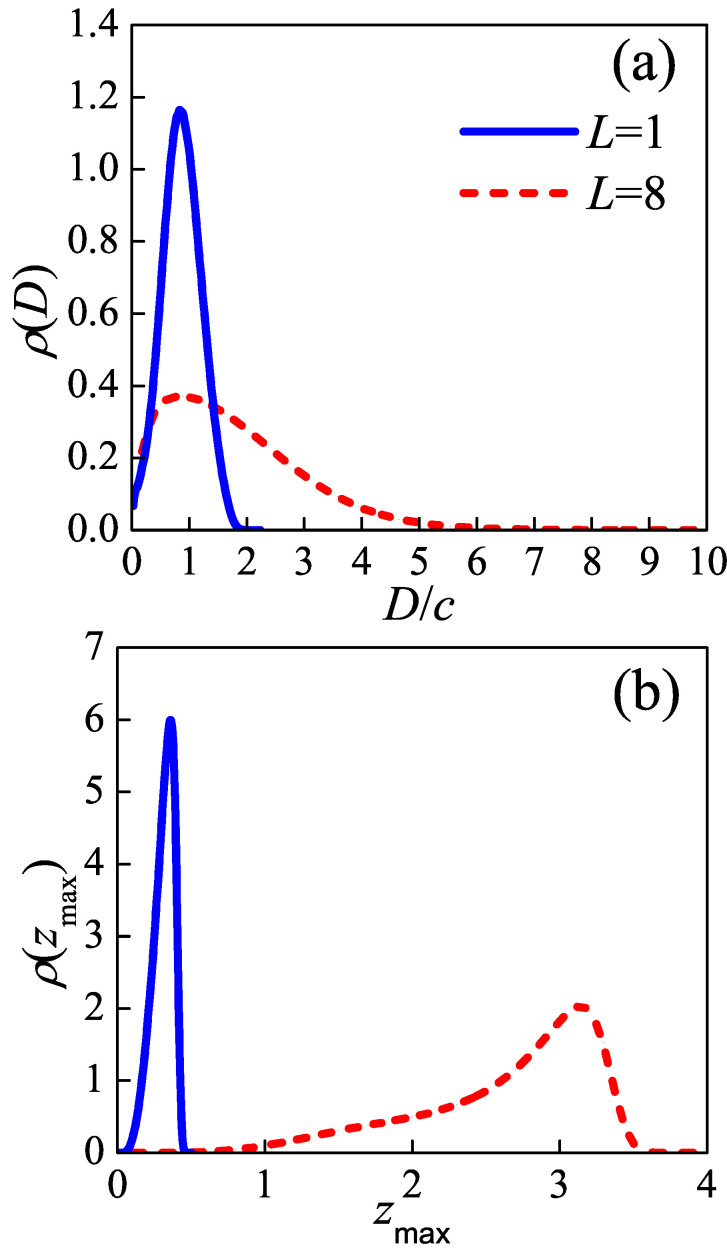
The distribution profile of (**a**) the distance between two injection points *D*, and (**b**) the distance between the lamellae edge and the highest point in the loop $z_{max}$. *D* is scaled by lattice constant *c*, and $z_{max}$ is scaled by L/2. The blue solid lines and red dash lines represent the tight fold (L=1) and loose loop (L=8), respectively.

The distribution of the distance between two injection points can be obtained by

(4) $$\rho \left(D\right)=\langle \delta \left[D-D\left\{R_{j}\left(s\right)\right\}\right]\rangle ,$$

as shown in Figure 2a. Both tight folds and loose loops display most probable distances between two injection points. The maximums of ρ(D) locate at $D^{*}/c=0.82$ for tight folds while 0.89 for loose loops. The plot of loose loop (L=8) takes a near-Gaussian form. If the loop length increase to infinity (L→∞), its distribution plot will degenerate to the right bank of a Gaussian curve. On the other hand, the plot of tight fold shows a sharp peak. In the short loop limit (L→0), loop has to re-enter the lamellar crystal before it can make any bendness, so the distribution function will take a δ-form whose peak appears at D/c∼L/c. Thus, the optimal distance between two injection points of loops is variable as loop length changing. However, in Muthukumar’s treatment, he used a constant cutoff value for *D* [16], which remains in the final result.

It is noteworthy that both the optimal distance between two injection points for tight fold and loose loop are near to and a bit smaller than one times the lattice constant, even though the loose loop length is longer than 17 times the lattice constant. $D^{*}/c$ of loose loop is slightly higher than $D^{*}/c$ of tight fold, although the length of the former is as eight times as long as the latter. The distribution of tight folds is only in the range D/c∈[0,2.2], because they are too short to reach a re-entry point exceeding two times the lattice constant. The distance between two injection points distributes centrally in the range no further than four times the lattice constant. These results provide evidence for the adjacent re-entry model.

The difference between interfaces formed by tight folds and loose loops could also be found from Figure 2b, which plots the distribution of distance between lamellar edge and the furthest point in the loop, i.e.,

(5) $$\rho \left(z_{max}\right)=\langle \delta \left[z_{max}-z_{max}\left\{R_{j}\left(s\right)\right\}\right]\rangle .$$

Both two curves show a peak. The peak appears at $z_{max}=0.36$ for tight fold, while at a more distant location, i.e., $z_{max}=3.2$, for a loose loop. This agrees with the physical expectation that a longer loop could visit a further distance. Besides, the larger peak width for L=8 than that for L=1 means the crystal-amorphous interface formed by loose loops has a larger roughness than the interface formed by tight folds.

**Figure 3:**
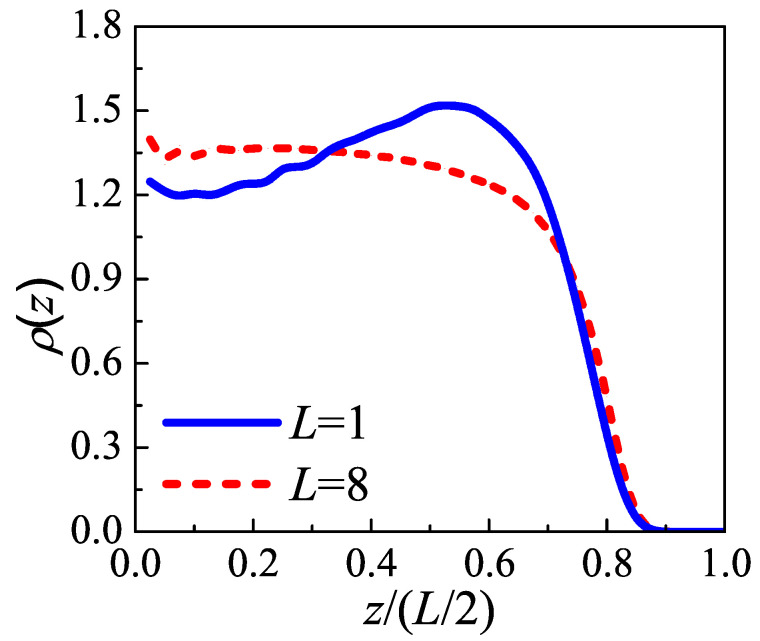
The density profiles versus the distance away from the surface. The distance away from the surface is scaled by half of loop length, i.e., L/2. The blue solid lines and red dash lines represent the tight fold (L=1) and loose loop (L=8), respectively.

The morphology and conformation of amorphous layer given by SCMFT is shown in Figure 3. The density distribution function along the normal direction of substrate is characterized by

(6) $$\rho \left(z\right)=\langle \frac{1}{L}\int_{0}^{L}\delta \left[z-z\left(s\right)\right]ds\rangle .$$

**Figure 4:**
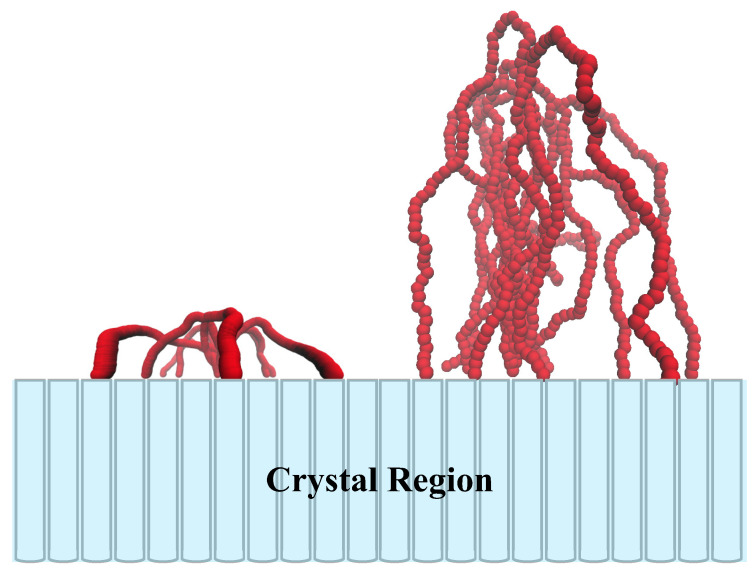
Conformational examples of tight folds (L=1, **left**) and loose loops (L=8, **right**) injecting outside the lamellar crystal. The conformations come from the important sampling by Monte Carlo in SCMFT. The blue bars represent the stems in crystal region.

In tight fold case, the density distribution increases moderately as *z* increasing at first, and shows a maximum at z/(L/2)=0.52. As *z* increasing further, the density distribution decays quickly, and finally reaches zero at z/(L/2)=0.9. The maximum can be explained by this consideration: a tight fold needs to reach back to the lamellae edge within a shorter length, thus it will preserve the conformation as an arc, and the density distribution of an arc has a natural maximum at a certain location away from the lamellae edge (as shown in the left panel of Figure 4). In the loose loop case, the density distribution hold a near-plateau form in a wide range, and as *z* increasing further, it will decrease monotonously, and reaches zero also at z/(L/2)=0.9. This indicates that the loose loops in amorphous layer take the “Bobby-pin”-like conformation: it consists of two straight legs and a sharp fold (as shown in the right panel of Figure 4). The sharper fold than tight fold case, just like an acute angle, results in the absence of maximum in density distribution profile. These are consistent with our results in Figure 2 and the analysis of Savage et al. [21], who proposed that the fold projecting farther out from the lamella to complete the fold would be less immobilized and in proximity to the crystal.

**Figure 5:**
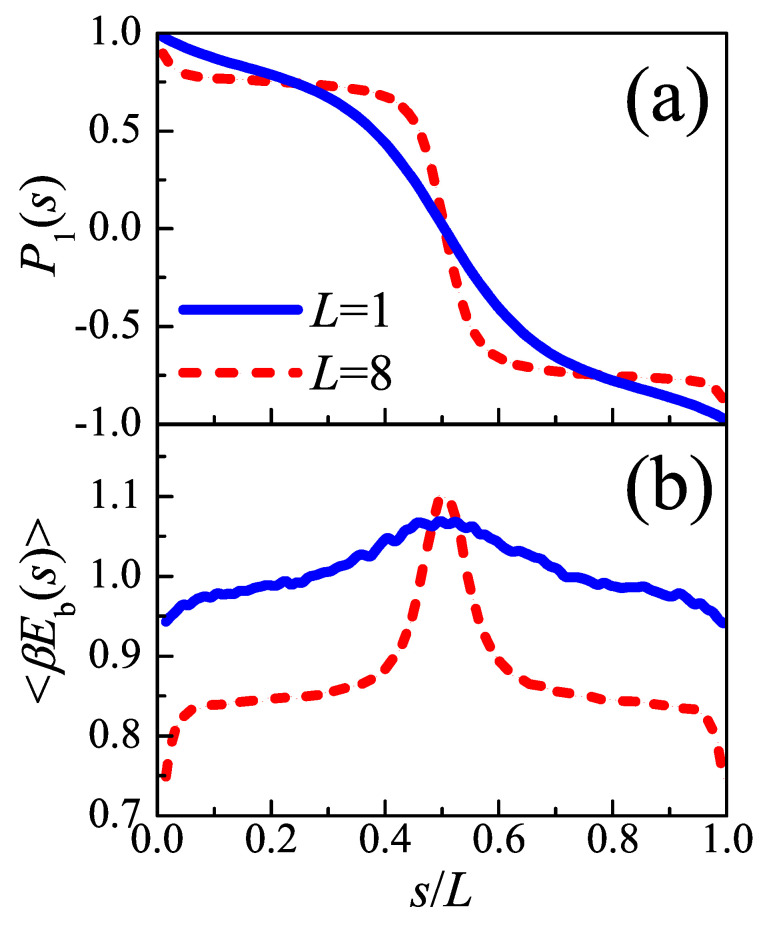
(**a**) The segment orientation functions and (**b**) the bending energy versus the coordinate along the loop chain. The blue solid lines and red dash lines represent the tight fold (L=1) and loose loop (L=8), respectively. *s* is scaled by loop length *L*.

To clarify the loop conformation in the amorphous layer more clearly, the segment orientation function $P_{1}\left(s\right)$ is computed by

(7) $$P_{1}\left(s\right)=\langle \cos\theta \left(s\right)\rangle ,$$

where θ(s) is the angle of tangent vector at *s* along the loop measured with respect to the direction perpendicular to the lamellae surface. For a perfect semi-circle conformation, the $P_{1}\left(s\right)$ should perform as a cosine curve. The results is shown in Figure 5a. The plots have centrosymmetry about point $\left(s/L=0.5,P_{1}\left(s\right)=0\right)$. The result of tight fold is more similar to a cosine curve, indicating that it hold a semi-circle conformation roughly. However, the behaviors of loose loops deviate from the semi-circle conformation, and reach a plateau after the drop at the very start. The plateau is maintained until about s/L=0.4, and then experiences a rapid drop. The segment orientation functions of both tight folds and loose loops have maximal slopes at s/L=0.5, because statistically speaking, the loops need to complete the fold at their midpoints. The segment orientation function of loose loops shows a clear drop which is absent in the tight folds case, which indicates the existence of a sharp turn in loose loop conformation, and the plateau corresponds to the straight legs of the “hobby-pin” conformation.

The average bending energy along a loop, defined by

(8) $$\beta E_{b}\left(s\right)=\frac{1}{2}{\left|\frac{du(s)}{ds}\right|}^{2},$$

is shown in Figure 5a. The curve of loose loop is lower than tight fold in most cases, because it is easy for a longer loop to release the stress caused by folding back to re-enter the lamellar crystal. However, the average bending energy at middle points of loose loop is larger than tight fold, proving the existence of a sharper fold in loose loops. The left and right wings of loose loop curve approximate to plateaus, related to the more straight conformation taken by the two halves of loose loop. Thus, the interfacial tension was accumulated on the edge of the amorphous layer formed by loose loops, while distributes more uniformly in the whole interface formed by tight loop.

### 3.2. Conformation of Polymeric Loop in Amorphous Layer

**Figure 6:**
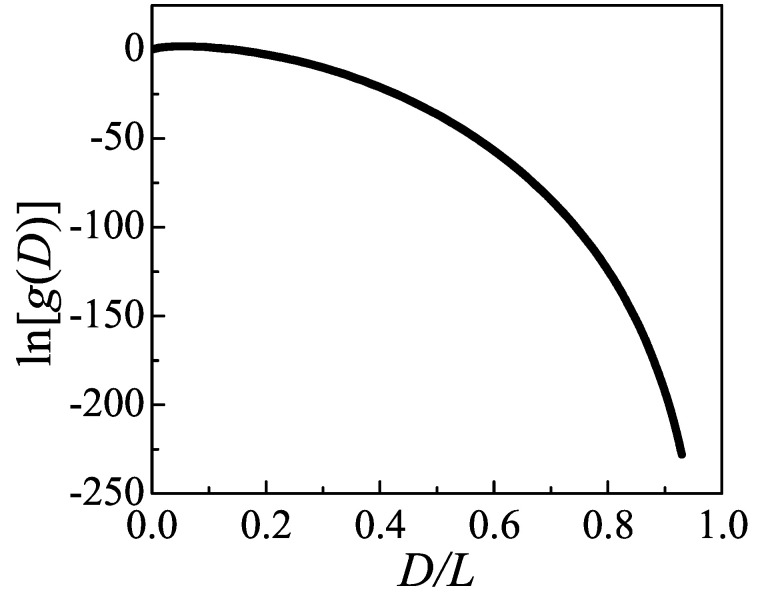
Density of states g(D) versus the distance between two injection points of an ideal loop. g(D) is plotted in a logarithmic plot, produced from the Wang–Landau Monte Carlo determination. *D* is scaled by loop length *L*. The absolute values of g(D) are meaningless, and g(D=0) is chosen to be zero.

The density of states for the distance between two injection points of loops, g(D), is shown in Figure 6, calculated in Equation (A15) by classic Wang–Landau algorithm. In general, g(D) declines as D/L increasing. This can be interpreted by considering the limit case that number of states is only 1 when the loop is fully stretched and pinned onto the lamellae edge. A maximum exists at D/L=0.06. If a propagator reaches back onto the lamellae edge after random walking in half-space by contour length *L*, its distance with original point is corresponding to the maximum value. The results of g(D) corresponds to the partition sum $g_{loop}\left(p\right)$ in Equation (18) in Ref. [16], in which an approximated formation was used.

**Figure 7:**
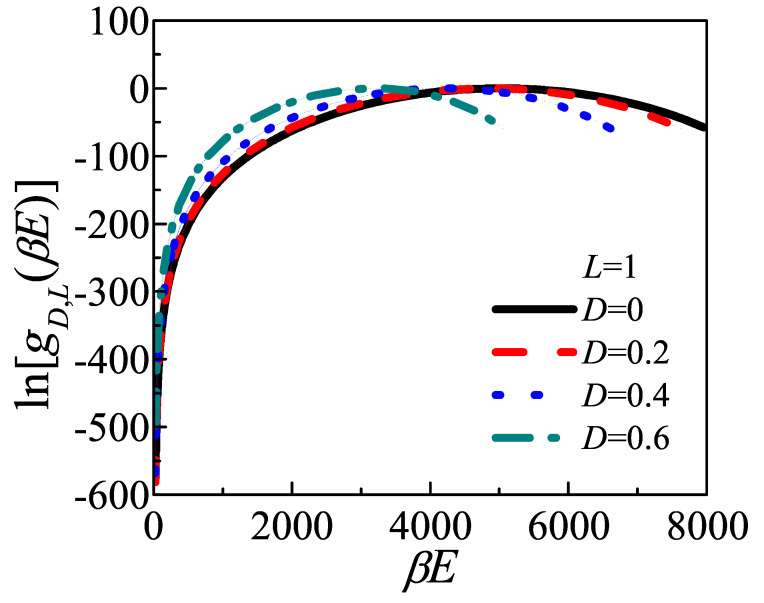
Examples of density of energy states $g_{D,L}\left(E\right)$ for a series given *D* when L=1 is plotted in a logarithmic plot, produced from the Wang–Landau Monte Carlo determination. Black solid line, red dash line, blue dot line, green dash dot line represent D=0,0.2,0.4,0.6, respectively. The absolute values of $g_{D,L}\left(E\right)$ and the relative value between different curves are both meaningless, and maximum of $g_{D,L}\left(E\right)$ curve is chosen to be zero. The range of energy in the case of smaller *D* is larger than that of larger *D*, because the ceiling energy of conformation with smaller *D* is higher.

Then the bending energy contribution, which has been ignored in Muthukumar’s theory, is taken into consideration in the present work. The density of energy states, $g_{D,L}\left(\beta E\right)$ is calculated by Equation (A21), and some examples in case of L=1 are shown in Figure 7. The modified Wang–Landau algorithm can has much better performance in the low energy states than the original one, as well as the precise global information in the whole energy space. Since the $g_{D,L}\left(\beta E\right)$ curves are plotted in a logarithmic plot, the density of energy states drops rapidly as energy approaching to ground state. This means the number of low energy states is quite few and is hard to visit by the classic Wang–Landau algorithm, and contributes large errors. These problems have been well solved by the present modified Wang–Landau algorithm.

**Figure 8:**
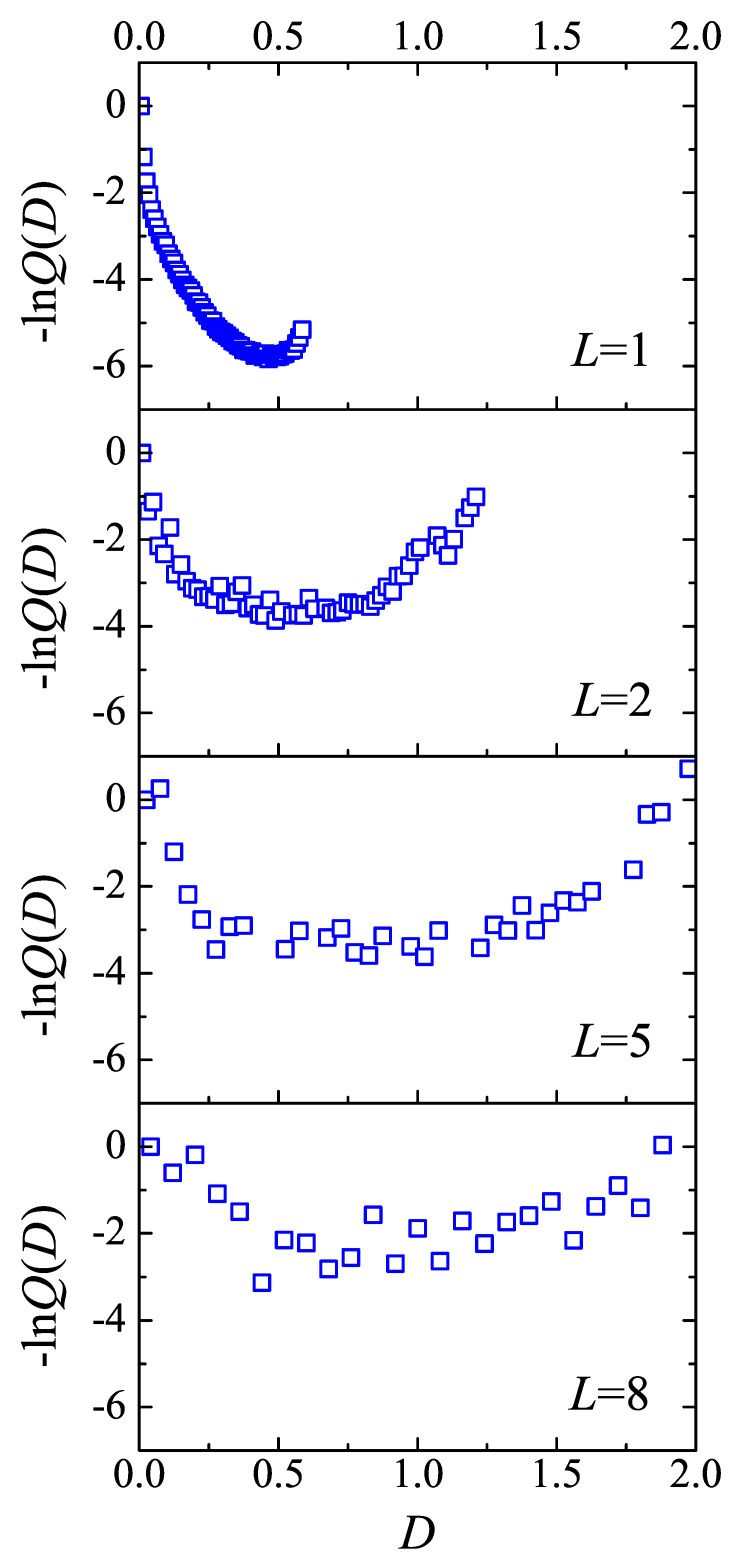
The value of term −lnQ(D) versus the distance between the two injection points of loops for loop length L=1,2,5,8. The absolute values of −lnQ(D), and the relative value between different curves, are both meaningless. −lnQ(D=0) of each curve is chosen to be zero.

Substituting above results into Equation (2) −lnQ as a function of *D* can be obtained, as shown in Figure 8. For tight fold case, there is a minimum at D=0.46. As *L* increasing, this minimum location moves toward left. These are the results of competition between enthalpy and entropy. For tight fold, the loop is short and entropy contribution is limited, thus the behavior of −lnQ(D) is dominated by energy. Tight folds search the conformation to minimize their energy. The two injection points, kept perpendicular to the lamellae edge. To minimizing the bending energy, the tight loops take the semi-circle conformation. Taking consideration of the excluded volume interaction, the distance between two injection points should be decreased. As a consequence the diameter of the semi-circle with perimeter *L*, i.e., 2L/π≈0.63L, is higher than 0.465, should be the upper bound of estimation of *D*. In loose loop condition, the entropy dominates structure of the amorphous layer. −lnQ(D) curves will approach to the opposite value of lng(D), thus the maximum location of g(D) curve, D/L=0.06, is the lower bound of estimation of the minimum location of −lnQ(D). Thus, the loop conformation in amorphous layer is the result of competition between entropy and energy.

### 3.3. The Effect of Density of Injection Points

**Figure 9:**
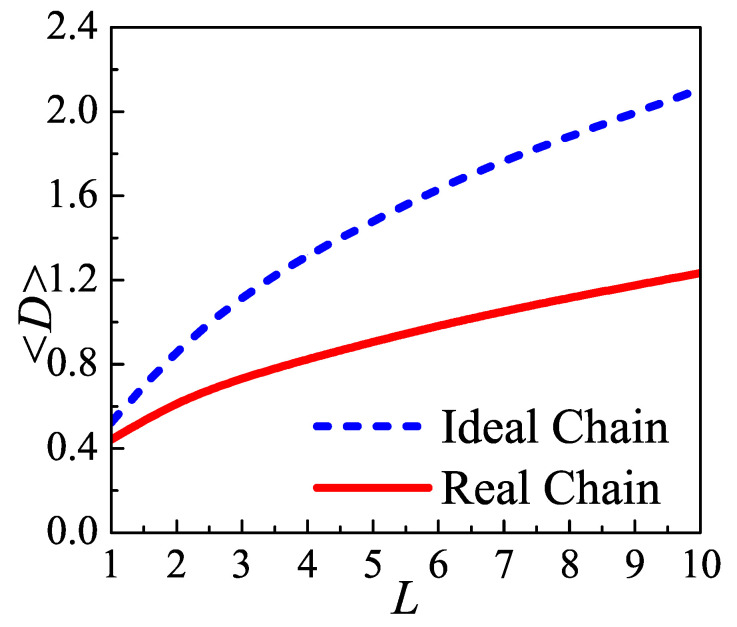
The optimal average distance between two injection points of loop versus its length for ideal/real chain. The blue dash line and red solid line correspond to ideal chain and real chain, respectively.density

Figure 9 presents the optimal average distance between two injection points of loops 〈D〉 as a function of loop length, computed by Equation (3). To reveal the effect of density of injection points, the optimal average distance 〈D〉 for various *L* is also given for ideal chain. For both ideal and real chains, the optimal average distance increases as loop length increasing. However, the slopes of these two curves are always much lower than 1, and decrease as *L* increasing. Namely, it is hard to gain a further 〈D〉 by simply increasing the loop length. The density of injection points induces loops to depress their value of 〈D〉. In consideration of lattice constant c=0.4, a first-neighbor fold needs loop length L≈1, a second-neighbor fold corresponds to L≈3, while a loop which strides only three crystal lattices and re-enters the lamellae edge needs L>10. The distance between two injection points is no more than several lattice constant even though the loop length increases to a large value. For a tight fold, it is too short to reach a distant re-entry point. For loose loops, if they re-enter the lamellae edge at a far point, they will constitute a thinner crystal-amorphous interface. With a given total number of monomers, the thinner interfacial thickness means the higher concentration. In this condition, loops tend to project themselves into the space further away from the lamellae edge to release the pressure, leading to the shrinkage of distance between two injection points. These also can be indicated by Figure 4. As a result, both tight folds and loose loops prefer adjacent re-entry. These results consists with a series of experiments [9,34] and simulations [38,39,40], which support that the adjacent re-entry occupy the major fractions of loop conformations.

**Figure 10:**
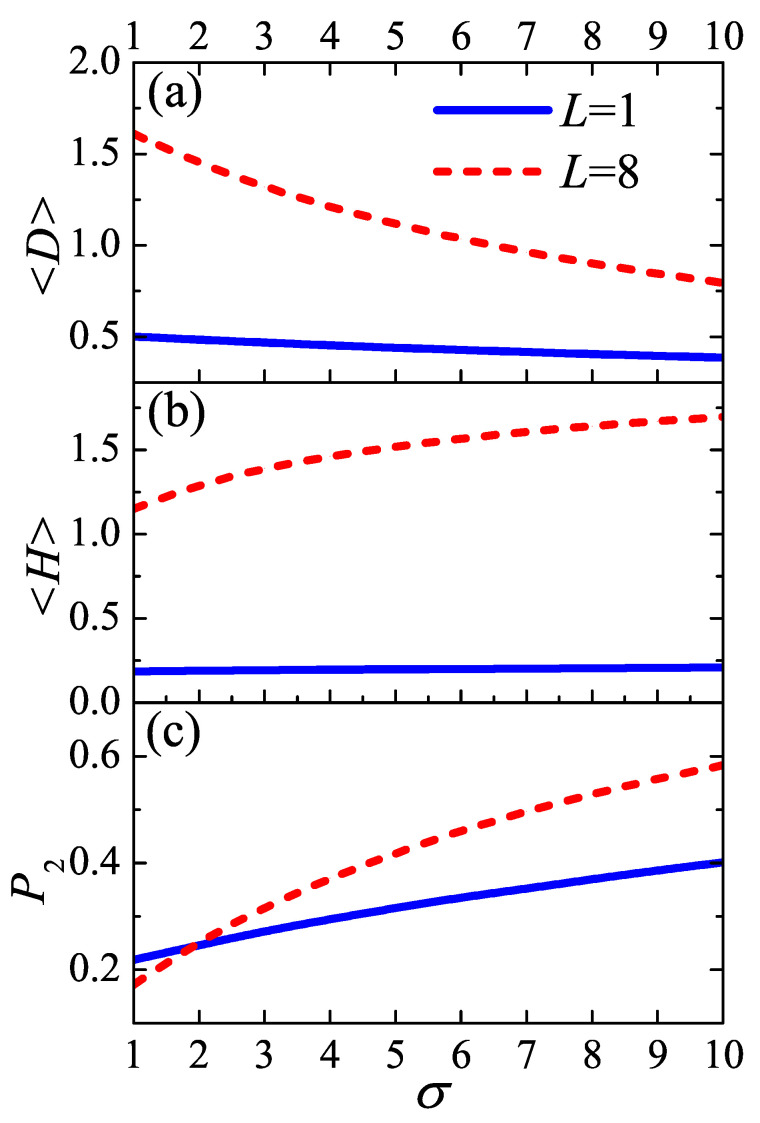
(**a**) The optimal average distance between two injection points of loop 〈D〉, (**b**) the average thickness 〈H〉, and (**c**) the segment orientation order parameter $P_{2}$, versus the density of injection points σ varying from 1 to 10. The blue solid lines and red dash lines correspond to tight fold (L=1) and loose loop (L=8), respectively. Higher value of $P_{2}$ is corresponding to higher ordered conformation.

For verifying our analysis, the optimal average distance between two injection points 〈D〉, the average height 〈H〉, and the segment orientation order parameter $P_{2}$ are displayed as functions of density of injection points for both tight fold and loose loop in Figure 10. The average height of one loop is defined by

(9) $$H=\frac{1}{L}\int_{0}^{L}z\left(s\right)ds,$$

where z(s) is the distance between the lamellae edge and a point along loop R(s). The segment orientation order parameter is defined by

(10) $$P_{2}=\frac{1}{L}\int_{0}^{L}\langle \frac{1}{2}\left(3\cos^{2}\theta \left(s\right)-1\right)\rangle ds,$$

were θ(s) is the angle between the bond vector and the normal direction of the substrate. As shown in Figure 10a, the optimal average distance decreases monotonically as the stems in polymer crystal becoming more compacted, which reflects the repulsive effect contributed by surrounding loops. The loose loop is much sensitive to the density of injection points than the tight folds. Both the loop length and the density of injection points vary in one order of magnitude, however, 〈D〉 varies in no more than 1 for both tight fold and loose loop and does not exceed the range of adjacent re-entry.

**Figure 11:**
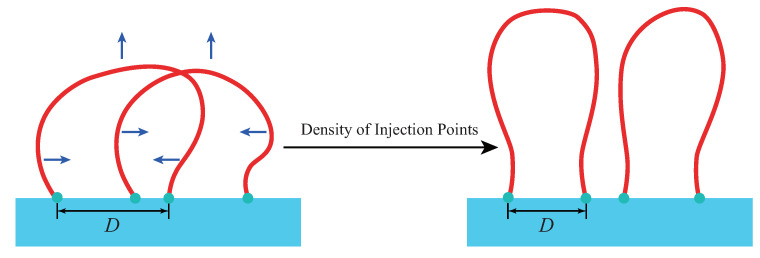
Schematic diagram of loop responding to the density of injection points. Two motion modes are presented: 1. the horizontal arrows represent the motion along the direction parallel to the lamellae edge, leading to the decreasing of 〈D〉; 2. the vertical arrows represent the motion along the direction of stems, leading to the increasing of 〈H〉.

The decreasing of 〈D〉 is accompanied with the increasing of average height 〈H〉 in Figure 10b. This is because a loop could stretch more into the amorphous region if its two injection points is nearer to each other. As σ increases, each loop in interface undergoes the confinement effect from the other loops nearby. The loops have two responsive ways to the excluded volume effect: (1) reducing their volume and shrinking in the direction parallel to the lamellae edge, via decreasing the distance between two injection points; (2) enlarging their asphericity and extending along the direction parallel to the stem vector, via spreading further away from the lamellae edge. These two motion modes are displayed in Figure 11. The combination of above two aspects results in the repulsion behavior among loops and improvement of order of amorphous layer, as shown in Figure 10c. In linear brush system, The ordering behavior induced by density of injection points [30,41,42] only involves the second motion mode. Finally, the density of injection points has larger influence on loose loop, which can be seen from the higher slope of loose loop in Figure 10b,c. These results demonstrate the behavior of interface during the phase transition from “Rotator” to crystal between which σ is the only difference [27].

## 4. Conclusions

In this paper, the local-exchange model for polymer amorphous layer, which is constituted mainly by folded loops, is proposed. In the amorphous layer, because the length of loops is much shorter than the total length of the chain and comparable with the Kuhn length, the semiflexible chain model was used instead of the Gaussian chain model which was adapted by previous theoretical studies on amorphous layer. Based on the worm-like chain model, the optimal distance between two injection points of a loop is determined by the balance of chain stiffness and conformational entropy. This model gives a possible interpretation for the origin of folding interface structure theoretically. The role of local-exchange behavior in polymer crystallization is emphasized in this model. The structure of amorphous layer formed by tight loops or loose loops has been investigated by SCMFT, respectively. The stems inside the lamellar crystal shift their locations to optimize the distance between two injection points. The result of optimal distance between two injection points for both tight and loose loop support the adjacent re-entry model. The effect of density of injection points is introduced to explain this behavior.

Besides, to balance the requirement for complete information in global energy space and the precise information in local low-energy states, a modified three-steps Wang–Landau algorithm has been developed to obtain the accurate solution of partition function for the worm-like chain.

## Abbreviations

The following abbreviations are used in this manuscript:

SCMFT	single-chain in mean-field Theory
SCFT	self-consistent field theory
WLC	Worm-like chain
AFM	Atom Force Microscopy

## Appendix A. Single-Chain in Mean-Field Theory

The Hamiltonian of a worm-like chain for a given conformation is given by

(A1) $$\begin{array}{c} \beta H_{0}\left[u(s)\right]=\frac{1}{2}\int_{0}^{L}ds{\left|\frac{du(s)}{ds}\right|}^{2}. \end{array}$$

The distribution function of a single conformation is

(A2) $$\begin{array}{ccc} P_{0}\left[R(s),u(s)\right] & = & \exp\left\{-\beta H_{0}\left[u(s)\right]\right\}\prod_{s}\left\{\delta \left[|u(s)|-1\right]\delta \left(u-\frac{dR(s)}{ds}\right)\right\}\delta \left[R_{z}\left(0\right)\right]\delta \left[R_{z}\left(L\right)\right]. \end{array}$$

The two δ functions in the bracket of {} in Equation (A2) implicate the constraint of smoothness and inextensibility. The last two δ functions mean the polymer loops inject into the amorphous region by crossing the surface of z=0.

The density operator is defined as

(A3) $$\begin{array}{c} \hat{\rho }\left(r,u\right)=\sum_{i=1}^{n}\int_{0}^{L}ds\delta \left[r-R_{i}\left(s\right)\right]\delta \left[u-u_{i}\left(s\right)\right]. \end{array}$$

The anisotropic excluded volume interaction is described by Onsager’s formula:

(A4) $$\begin{array}{c} \beta V\left(r,r^{\prime },u,u^{\prime }\right)=2da^{2}\delta \left(r-r^{\prime }\right)\left|u\times u^{\prime }\right|. \end{array}$$

The interaction between loop segments and crystal-amorphous interface are described by the impenetrable potential

(A5) $$\begin{array}{c} \beta V_{I}\left(r\right)=\left\{\begin{array}{cc} 0 & z>0\\ \infty & z<0 \end{array}\right.. \end{array}$$

After conventional derivation, the interfacial free energy could be written as

(A6) $$\begin{array}{c} \beta F\left[\omega \right]\equiv -\frac{1}{2}\int drdudu^{\prime }\frac{\omega \left(r,u\right)\omega \left(r,u^{\prime }\right)}{2da^{2}\left|u\times u^{\prime }\right|}-n\ln Q\left[\omega \right], \end{array}$$

and the single chain partition function is

(A7) $$\begin{array}{ccc} Q[\omega ] & \equiv & \int D\left\{R\left(s\right),u\left(s\right)\right\}\prod_{s}\left\{\delta \left[|u\left(s\right)|-1\right]\delta \left(u\left(s\right)-\frac{\partial R(s)}{\partial s}\right)\right\}\delta \left[R_{z}\left(0\right)\right]\delta \left[R_{z}\left(L\right)\right]\\ & & \times \exp\left\{-\beta H_{0}\left[u(s)\right]-\int ds\left\{\omega \left[R(s),u(s)\right]+V_{I}\left[R(s)\right]\right\}\right\}, \end{array}$$

Performing mean field approximation δF/δω=0, we have the self-consistent field equation

(A8) $$\begin{array}{ccc} \omega (r,u) & = & 2da^{2}\int du^{\prime }\left|u\times u^{\prime }\right|\rho \left(r,u^{\prime }\right), \end{array}$$

where

(A9) $$\begin{array}{ccc} \rho (r,u) & \equiv & \langle \hat{\rho }\left(r,u\right)\rangle \\ & = & \frac{LA\sigma }{Q}\int D\left\{R\left(s\right),u\left(s\right)\right\}\prod_{s}\left\{\delta \left[|u\left(s\right)|-1\right]\delta \left[u-\frac{dR(s)}{ds}\right]\right\}\delta \left[R_{z}\left(0\right)\right]\delta \left[R_{z}\left(1\right)\right]\\ & & \times \int_{0}^{1}ds\delta \left[r-R\left(s\right)\right]\delta \left[u-u\left(s\right)\right]\exp\left\{-\beta H_{0}\left[u(s)\right]-\int ds\left\{\omega \left[R(s),u(s)\right]+V_{I}\left[R(s)\right]\right\}\right\}. \end{array}$$

It should be mentioned that *d* here is a parameter from the Onsager interaction which characterizes the thickness of the polymer. In present model, *d* is not an independent variable. The product of excluded volume, *v* and the density of injection points, σ is the independent variable. Here, the excluded volume of the segment is defined by $v=2da^{2}$. Therefore, a reduced density of injection points can be defined σ→vσ.

## Appendix B. Calculation of the Single Chain Partition Function

In this paper, a symbol with angle brackets, 〈A〉, denotes the average of a physical quantity *A* in a canonical ensemble,

(A10) $$\langle A\left(L\right)\rangle =\frac{\int d(\cdot )A(\cdot )\exp[-\beta \varepsilon (L,\cdot )]}{\int d(\cdot )\exp[-\beta \varepsilon (L,\cdot )]},$$

where the abbreviation (·) is used for all variables that describe the positions of all monomers of the loop polymer. The total energy ε(L,·) contains three terms corresponding to the bending energy, the excluded volume effect, and the hard-wall potential of a specified conformation (·), respectively, i.e.,

(A11) $$\varepsilon \left(L,\cdot \right)=\beta H_{0}\left[u(s)\right]+\int ds\left\{\omega \left[R(s),u(s)\right]+V_{I}\left[R(s)\right]\right\}.$$

The denominator in Equation (A10) is partition function,

(A12) Q=∫d(·)exp[−βε(L,·)].

For any given loop configuration described by the coordinates (·), it has a distance between the two injection points ζ(·),

(A13) ζ(·)=|R(L)−R(0)|.

In fact, during a MC production run with statistical weight being W(D), the measured histogram of visited *D* is related to

(A14) H(D)=∫d(·)W[ζ(·)]δ[D−ζ(·)],

where δ is Dirac’s delta function. Because the density of states for *D* is defined by

(A15) g(D)=∫d(·)δ[D−ζ(·)],

in a production run where W(ζ)=1/g(ζ) is used as a statistical weight, H(D) reaches a *D*-independent constant after adequate statistics. (The advantage of using 1/g(ζ) as the transition probability in a MC simulation is such that the simulated polymer goes through all the *D* bins many times in a MC run.) We can rewrite Equation (A10)

(A16) $$\langle A\left(L\right)\rangle =\frac{\int_{0}^{D_{m}}dDg\left(D\right)\langle A\left(D,L\right)\rangle }{\int_{0}^{D_{m}}dDg\left(D\right)\langle Q\left(D,L\right)\rangle },$$

where $D_{m}$ is the upper bound of *D*, W(ζ)=1/g(ζ), and

(A17) $$\langle A\left(D,L\right)\rangle =\frac{\int d(\cdot )W[\zeta (\cdot )]A(\cdot )\exp[-\beta \varepsilon (L,\cdot )]\delta [D-\zeta (\cdot )]}{\int d(\cdot )W[\zeta (\cdot )]\delta [D-\zeta (\cdot )]},$$

(A18) $$\langle Q\left(D,L\right)\rangle =\frac{\int d(\cdot )W[\zeta (\cdot )]\exp[-\beta \varepsilon (L,\cdot )]\delta [D-\zeta (\cdot )]}{\int d(\cdot )W[\zeta (\cdot )]\delta [D-\zeta (\cdot )]},$$

is the partition function for given *D* and *L*. The partition function for *D* is embodied in Equation (A16)

(A19) Q(D)=g(D)〈Q(D,L)〉.

It is formidable task to compute it using a simple sampling. To overcome this difficulty, we use the Wang–Landau algorithm to compute Q(D,L) as well:

(A20) $$\langle Q\left(D,L\right)\rangle =\frac{\int_{0}^{E_{m}}dEg_{D,L}\left(E\right)\exp\left(-\beta E\right)}{\int_{0}^{E_{m}}dEg_{D,L}\left(E\right)},$$

where

(A21) $$g_{D,L}\left(E\right)=\int d\left(\cdot \right)\delta \left[E-\varepsilon_{D,L}\left(\cdot \right)\right].$$
